# Socio-Cultural Values Are Risk Factors for COVID-19-Related Mortality

**DOI:** 10.1177/10693971211067050

**Published:** 2022-04

**Authors:** Ansgar D. Endress

**Affiliations:** 1 Department of Psychology, 4895 City, University of London, UK

**Keywords:** socio-cultural values, COVID-19, environmental health, risk factors

## Abstract

To assess whether socio-cultural values are population-level risk factors for health, I sought to predict COVID-19-related mortality between 2 weeks and 6 months after the first COVID-19-related death in a country based on values extracted from the World Values Survey for different country sets, after controlling for various confounding variables. COVID-19-related mortality was increased in countries endorsing political participation but decreased in countries with greater trust in institutions and materialistic orientations. The values were specific to COVID-19-related mortality, did not predict general health outcomes, and values predicting increased COVID-19-related mortality predicted decreased mortality from other outcomes (e.g., environmental-related mortality).

## Introduction

Individual behavior accounts for a third of premature loss of life (Stanaway et al., 2018). It can affect health outcomes directly (e.g., lifestyle affects cardiovascular health; Stanaway et al., 2018; Elmer et al., 2006) or through general behavioral traits (e.g., childhood impulsivity predicts later health; Schlam et al., 2013).

Some health-related behaviors are culturally determined. For example, some food taboos are toxicity-related (Henrich & Henrich, 2010), diet affects cardiovascular disease prevalence across ethnic groups (Volgman et al., 2018), and intra-couple power relations predict HIV prevalence in young women (Jewkes et al., 2010). This raises the possibility that, at the population level, socio-cultural values might be risk factors for disease susceptibility, by promoting both health-related behaviors and institutional structures that affect health.

Here, I test this possibility by seeking to identify risk factors for country-level mortality from the recent coronavirus disease 2019 (COVID-19) outbreak based on socio-cultural values extracted from the World Values Survey (WVS; Inglehart et al., 2014). The WVS is a global set of national surveys tracking values and beliefs; it comprises questions assessing, among many other values, attitudes towards democracy, gender equality, religion and economic inequality.

Such values might plausibly be risk factors for COVID-19-related mortality, though the results are somewhat inconsistent. For example, in the United States, political affiliation predicts compliance with COVID-19 containment measures (Grossman et al., 2020; Hsiehchen et al., 2020), which, in turn, likely leads to mortality differences. Conversely, countries with “tighter” cultures with more stringent social norms have reduced COVID-19-related mortality compared to “looser” cultures (Gelfand et al., 2021), even though cultural tightness is associated with more conservative leanings (Harrington & Gelfand, 2014). Other associations between COVID-19-related outcomes and cultural values have been similarly controversial. Across countries, the spread of COVID-19 was accelerated in countries with more social trust, at least in the early phases of the pandemic (Min, 2020); however, across *counties* in the United States, social capital (and thus social trust) had a protective effect against COVID-19 (Makridis & Wu, 2021), while still other studies found no relationship between social trust and acceptance of COVID-19-related measures and restrictions (Romano et al., 2021).

In addition to individual health-related behavior, socio-cultural values might also affect institutional responses. For example, it might be harder to constrain individual liberties in societies with stronger democratic norms than in more authoritarian societies; accordingly, societies with stronger democratic norms were slower to enact stringent containment measures in response to the COVID-19 pandemic (Sebhatu et al., 2020).

However, such earlier studies predicted COVID-19-related outcomes based on established constructs such as social trust or cultural tightness (Gelfand et al., 2021; Makridis & Wu, 2021; Min, 2020), some of which have been linked to historic disease prevalence (Gelfand et al., 2011; Harrington & Gelfand, 2014). As a result, it is unclear if these constructs are the best predictors of COVID-19-related outcomes, and to what extent these constructs are specifically associated with COVID-19-related outcomes rather than with general health outcomes that happen to be associated with COVID-19-related outcomes *as well*. To address these issues, I seek to identify risk factors of COVID-19-related mortality in a data-driven fashion based on all questions in the WVS. I then show that these values are not associated with general health outcomes, and that values associated with better COVID-19-related outcomes are associated with worse outcomes for other health conditions.

To foreshadow the results, I found that COVID-19-related mortality is increased in countries endorsing values related to political participation, but decreased in countries with more trust in institutions and materialistic orientations. I then asked whether these values were specific to COVID-19-related mortality, or whether they were simply associated with health outcomes *in general*. I assessed the specificity of the WVS predictors in two ways. First, I compared the values associated with COVID-19-related mortality to those associated with a general measure of health (healthy life expectancy at age 60) but could not identify any reliable predictors. Second, I compared the WVS predictors of COVID-19-related mortality with those of mortality in a domain where institutional structures are important in addition to individual behavior: environmental-related mortality.

To assess the reliability of these results, I compare them across different country sets, namely, all countries for which data is available, Upper-Middle-Income and High-Income Economies as defined by the World Bank (economies with a GNI per capita between $4046 and $12,535 and above $12,535, respectively) and Advanced Economies as defined by the International Monetary Fund, respectively (see SM1.1.7 for the country lists).

The types of risk factors for COVID-19-related outcomes make it difficult to deduce specific causal pathways that lead from these risk factors to health outcomes. In fact, COVID-19-related outcomes might be affected by cultural values in at least three, potentially interacting, ways. First, cultural values might promote behaviors in the general population that affect COVID-19-related outcomes as a result. Second, given that institutions such as governments consist of humans, cultural values might affect governmental actions, which might affect COVID-19-related outcomes in turn. Third, given that institutions are also created by humans, institutional structures are likely influenced by cultural values as well, which might also affect COVID-19-related outcomes. As these influences are difficult to tease apart in the absence of targeted intervention studies, the analyses below will be limited to identifying risk factors for COVID-19-related outcomes; in the Discussion, I will elaborate on different possible causal pathways in more detail.

## Methods

Full methods and data sources are given in SM1. Confirmed COVID-19-related deaths were downloaded from the Johns Hopkins University Center for Systems Science and Engineering; other data were obtained from the World Bank and the World Health Organization. I used confirmed COVID-19-related deaths as the primary health outcome because they are less affected by the testing regime than other outcomes. I calculated predictions using both linear and k-nearest neighbor models but will focus on the linear models as they performed somewhat better.

I identified mortality-related values in two steps. First, after preprocessing (see SM1), I removed WVS predictors that were strongly correlated with one another and kept, for each set of correlated WVS predictors, the predictor available for the largest number of countries. Second, I selected those WVS predictors whose absolute correlation with COVID-19-related mortality exceeded that of 95% of the WVS predictors, and among these WVS predictors, the three predictors with the highest absolute correlation.

This predictor selection procedure was necessary because dimensionality reduction techniques such as principal component analysis are not applicable because not all questions were administered in all countries. In fact, earlier studies using principal component analysis with the WVS did so at the cost of significantly curtailing the number of predictors so that all predictors would be available for all countries (e.g., 43 predictors for 43 societies in Inglehart, 1997, p. 82, or just 10 predictors for 81 societies, Inglehart & Welzel, 2005, p. 49). Using dimensionality reduction techniques thus creates a dilemma between restricting coverage by reducing the number of countries or introducing an analytic bias by choosing predictors the analyst might expect to reflect factors that are chosen a priori. Further, the specific components of a principal component analysis are somewhat arbitrary in that they depend on the specific questions, and sometimes investigators decide to sub-divide components for theoretical reasons (e.g., Kaasa, 2021). Similar dilemmas also arise when applying “wrapper” approaches (in Kohavi and John’s (1997) terminology) that evaluate the contributions of individual predictors to the overall fit of a model because they also require all predictors to be available for all countries. To avoid such dilemmas, I thus opted for a data-driven selection procedure (see, e.g., Hall, 1998; Hall & Holmes, 2003; Yu & Liu, 2003 for other correlation-based filters for predictor selection) and will use correlations with other predictors as a makeshift component analysis. The main drawback of this approach is that it is difficult to investigate interaction effects.

I then asked whether a linear model incorporating these WVS predictors would predict mortality better than a baseline model using only the covariates above. To ascertain that these predictions were not an artifact of the countries on which they were based, I first fitted the models to 50 random subsets of 80% of the countries in each country set and evaluated the predictions for the remaining 20%. Finally, I sought to predict mortality for the full dataset. In all analyses, I controlled for (1) per capita GDP, (2) the proportion of the population older than 65, (3) the proportion of the population living in urban areas, (4) the prevalence of tobacco smoking, (5) the proportion of-out-of-pocket health expenditure, (6) the (positive or negative) delay of the first government containment measure since the first confirmed COVID-19-related death in a country, and, for High-Income and Advanced Economies, (7) the prevalence of overweight. In SM3.2, I also report analyses controlling for government effectiveness and the rule of law (Kaufmann et al., 2010). As both indicators were strongly correlated with per capita GDP (government effectiveness: *ρ* = .83; rule of law: *ρ* = .78), I decided not to include these predictors into the model to avoid excluding countries for which these predictors were not available. As shown in SM3.2, the results are very similar when these predictors are included.

For each country set, I calculated four-weekly snapshots of COVID-19-related mortality, starting 14 days after the first confirmed death in each country and ending 182 days (6 months) after the first confirmed death. As shown in Table 1, depending on the snapshot date, complete data was available for 67–76 countries (all countries), 46–56 countries (Upper-Middle and High-Income Economies), 21–23 countries (Upper-Middle Income Economies), 21–28 countries (High-Income Economies), and 18–23 countries (Advanced Economies) countries.

**Table 1.** Model diagnostics for snapshots for different country sets and delays from the first confirmed COVID-19-related death in each country. ${\bar{R}}^{2}$s are adjusted coefficients of determination, ρ’s partial correlation coefficients after controlling for various covariates (see main text), and β’s coefficients in the linear models. Prediction error reductions are relative to baseline models predicting COVID-19-related mortality based on various covariates (see main text) but no predictors from the World Values Survey (WVS predictors). For full models (using all countries in a country set), prediction error reductions were evaluated by comparing the model fits to those in the baseline models using *F* tests. For cross-validation models, prediction error reductions were evaluated by comparing the set of error reductions across cross-validation runs against zero using signed rank tests. While WVS predictors were extracted to predict COVID-19-related mortality, model fits and parameters were calculated to predict both COVID-19-related mortality (black rows) and environmental-related mortality (gray rows). Missing parameter estimates indicate that the corresponding WVS predictors were not selected for a snapshot rather than that they were not correlated with mortality. See Table S1 for similar analyses in which government effectiveness and the rule of law are included as covariates.

			Delay since first confirmed COVID-19-related death in each country						
Measure	Predictor	# countries	14	42	70	98	126	154	182
**Model diagnostics (all countries)**									
${\bar{R}}^{2}$		67–76	0.241	0.638	0.63	0.524	0.382	0.345	0.344
% reduction prediction error versus baseline (COVID-19—full model)		67–76	4.22*	18.9***	22***	20.4***	17.8***	17.3***	17.2***
% reduction prediction error versus baseline (COVID-19—cross valid.)		67–76	0.504	11.8***	14.6***	13.2***	13.7***	9.75***	9.05***
*% reduction prediction error* vs. *baseline (env. DALYs*—*full model)*		*67–76*	*1.86*	9.39**	10.1**	10.1**	10.1**	10.1**	9.39**
**Model diagnostics (upper-middle and high income countries)**									
${\bar{R}}^{2}$		46–56	0.2	0.443	0.391	0.318	0.257	0.291	0.341
% reduction prediction error versus baseline (COVID-19—full model)		46–56	17.5**	20.6***	18.9***	18***	17.1**	19.5***	22.7***
% reduction prediction error versus baseline (COVID-19—cross valid.)		46–56	7.71***	8.62***	5.9**	6.07**	5.46**	10***	11.6***
*% Reduction prediction error* vs. *baseline (env. DALYs*—*full model)*		*46–56*	*3.3*	*6.2*	*6.2*	*6.2*	*0.742*	*0.742*	*0.742*
**Model diagnostics (upper-middle income countries)**									
${\bar{R}}^{2}$		21–23	0.434	0.452	0.553	0.526	0.628	0.672	0.661
% reduction prediction error versus baseline (COVID-19—full model)		21–23	35.8*	37.7*	38.4**	31.1**	47.2**	51.7***	49.6***
% reduction prediction error versus baseline (COVID-19—cross valid.)		21–23	10.5**	11.3**	24.5***	19.4***	22.4***	26.8***	29***
*% reduction prediction error* vs. *baseline (env. DALYs*—*full model)*		*21–23*	*4.89*	*24.3*	*10.5*	*0.00629*	*7.39*	*7.39*	*7.39*
**Model diagnostics (high income countries)**									
${\bar{R}}^{2}$		21–28	0.581	0.719	0.423	0.406	0.399	0.51	0.53
% reduction prediction error versus baseline (COVID-19—full model)		21–28	42.7*	46.2**	22.4	24.5	25.3	32.2**	33.1**
% reduction prediction error versus baseline (COVID-19—cross valid.)		21–28	11.1**	10.8**	−33.8**	−12.7	−49.1***	9.21**	15***
*% reduction prediction error* vs. *baseline (env. DALYs—full model)*		*21–28*	*18.6*	45.8**	*21.3*	*21.3*	*21.3*	*16.5*	*16.5*
**Model diagnostics (advanced economies)**									
${\bar{R}}^{2}$		18–23	0.465	0.686	0.579	0.674	0.655	0.665	0.574
% reduction prediction error versus baseline (COVID-19—full model)		18–23	18.3	46.9*	31.5*	41.4**	40.2**	39.7**	27.8
% reduction prediction error versus baseline (COVID-19—cross valid.)		18–23	−48.1***	12.5*	4.99**	10.8*	14.1***	3.84	−27.2*
*% reduction prediction error* vs. *baseline (env. DALYs*—*full model)*		*18–23*	*9.51*	*1.7*	*9.18*	*9.43*	*9.43*	*15.7*	*24.2*
**Model parameters (all countries)**									
*ρ*/*β* (COVID-19)	Aims of country: protecting freedom of speech	67–76		0.401***/0.466**	0.437***/0.577***	0.435***/0.626***	0.412***/0.648**	0.399***/0.624**	0.396***/0.553**
*β (env. DALYs)*	*Aims of country: protecting freedom of speech*	*67–76*		−0.112***	−0.114***	−0.115***	−0.115***	−0.114***	−0.112***
*ρ*/*β* (COVID-19)	Confidence in major companies	67–76			−0.348**/−0.348*	−0.35**/−0.379*	−0.346**/−0.426*	−0.341**/−0.428*	
*β (env. DALYs)*	*Confidence in major companies*	*67–76*			*−0.031 9*	*−0.031*	*−0.031*	*−0.031 2*	
*ρ*/*β* (COVID-19)	Confidence in press	67–76		−0.373***/−0.328					−0.356**/−0.355
*β (env. DALYs)*	*Confidence in press*	*67–76*		*−0.006 04*					*−0.005 58*
*ρ*/*β* (COVID-19)	Religious person	67–76		0.408***/0.576**	0.423***/0.596**	0.388***/0.544**	0.344**/0.492*	0.348**/0.513*	0.376***/0.589*
*β (env. DALYs)*	*Religious person*	*67–76*		*0.057 5*	*0.050 5*	*0.049 8*	*0.049 8*	*0.049 7*	*0.056 6*
**Model parameters (upper-middle and high income countries)**									
*ρ*/*β* (COVID-19)	Aims of country: protecting freedom of speech	46–56		0.486***/0.602**	0.44***/0.574*	0.422**/0.581*			
*β (env. DALYs)*	*Aims of country: protecting freedom of speech*	*46–56*		−0.083 7*	−0.083 7*	−0.083 7*			
*ρ*/*β* (COVID-19)	Confidence in major companies	46–56		−0.378**/*−*0.332	−0.404**/*−*0.459	−0.406**/*−*0.525	−0.438***/*−*0.482	−0.465***/*−*0.51	−0.5***/*−*0.529
*β (env. DALYs)*	*Confidence in major companies*	*46–56*		*−0.030 7*	*−0.030 7*	*−0.030 7*	*−0.023*	*−0.023*	*−0.023*
*ρ*/*β* (COVID-19)	Confidence in press	46–56		−0.468***/*−*0.305	−0.435***/*−*0.256	−0.393**/*−*0.204	−0.406**/*−*0.362	−0.437***/*−*0.402	−0.491***/*−*0.487*
*β (env. DALYs)*	*Confidence in press*	*46–56*		*0.007 41*	*0.007 41*	*0.007 41*	*0.029 3*	*0.029 3*	*0.029 3*
*ρ*/*β* (COVID-19)	Important for person: politics	46–56	−0.393**/*−*0.693*				−0.388**/*−*0.438	−0.412**/*−*0.468	−0.42**/*−*0.436
*β (env. DALYs)*	*Important for person: politics*	*46–56*	*0.010 7*				*−0.013 5*	*−0.013 5*	*−0.013 5*
**Model parameters (upper-middle income countries)**									
*ρ*/*β* (COVID-19)	Important for person: leisure time	21–23	0.557**/0.595	0.496*/0.526					
*β (env. DALYs)*	*Important for person: leisure time*	*21–23*	*−0.108*	*−0.129*					
*ρ*/*β* (COVID-19)	Important for person: politics	21–23					−0.591**/*−*0.339	−0.664***/*−*0.518	−0.683***/*−*0.567
*β (env. DALYs)*	*Important for person: politics*	*21–23*					*−0.013 5*	*−0.013 5*	*−0.013 5*
*ρ*/*β* (COVID-19)	Justifiable: divorce	21–23					0.624**/0.588	0.679***/0.692	0.702***/0.748
*β (env. DALYs)*	*Justifiable: divorce*	*21–23*					*0.109*	*0.109*	*0.109*
*ρ*/*β* (COVID-19)	Attended lawful peaceful demonstrations	21–23		0.516*/0.825	0.49*/0.892				
*β (env. DALYs)*	*Attended lawful peaceful demonstrations*	*21–23*		*0.146*	*0.14*				
*ρ*/*β* (COVID-19)	Good political system: having experts make decisions	21–23		0.485*/0.39	0.583**/0.741*	0.635**/0.98**	0.692***/0.774*	0.702***/0.721*	0.671***/0.573
*β (env. DALYs)*	*Good political system: having experts make decisions*	*21–23*		*0.025*	*−0.003 77*	*0.002 45*	*−0.051 9*	*−0.051 9*	*−0.051 9*
**Model parameters (high income countries)**									
*ρ*/*β* (COVID-19)	Aims of country: maintaining order in nation	21–28			−0.384*/*−*0.219	−0.422*/*−*0.244	−0.424*/*−*0.256	−0.412*/*−*0.388	−0.394*/*−*0.333
*β (env. DALYs)*	*Aims of country: maintaining order in nation*	*21–28*			*0.081 5*	*0.081 5*	*0.081 5*	0.083 8*	0.083 8*
*ρ*/*β* (COVID-19)	Essential characteristics of democracy: civil rights protect people’s liberty against oppression	21–28		0.468*/1.06*	0.488*/0.995*	0.457*/1.01*	0.444*/1.03*		
*β (env. DALYs)*	*Essential characteristics of democracy: civil rights protect people’s liberty against oppression*	*21–28*		0.199**	*0.117*	*0.117*	*0.117*		
*ρ*/*β* (COVID-19)	Essential characteristics of democracy: people receive state aid for unemployment	21–28	0.646***/1.18*	0.448*/0.377					
*β (env. DALYs)*	*Essential characteristics of democracy: people receive state aid for unemployment*	*21–28*	*−0.107*	−0.149**					
*ρ*/*β* (COVID-19)	Important child qualities: religious faith	21–28			0.447*/0.649	0.487**/0.874	0.496**/0.97	0.512**/0.703	0.517**/0.682
*β (env. DALYs)*	*Important child qualities: religious faith*	*21–28*			*0.083 2*	*0.083 2*	*0.083 2*	*0.018 8*	*0.018 8*
*ρ*/*β* (COVID-19)	Signed a petition	21–28	−0.484**/*−*0.997	−0.447*/*−*0.535					
*β (env. DALYs)*	*Signed a petition*	*21–28*	*−0.099 9*	−0.207**					
*ρ*/*β* (COVID-19)	Good political system: having army rule	21–28						0.45*/0.617	0.48**/0.651*
*β (env. DALYs)*	*Good political system: having army rule*	*21–28*						*0.064 3*	*0.064 3*
**Model parameters (advanced economies)**									
*ρ*/*β* (COVID-19)	Aims of country: protecting freedom of speech	18–23		0.59**/0.799		0.498*/1.04*	0.496*/1.06*		
*β (env. DALYs)*	*Aims of country: protecting freedom of speech*	*18–23*		*−0.003 5*		*0.019 5*	*0.019 5*		
*ρ*/*β* (COVID-19)	Aims of country: maintaining order in nation	18–23			−0.608**/*−*0.542	−0.621**/*−*0.694*	−0.611**/*−*0.71*	−0.588**/0.557	−0.566**/*−*0.324
*β (env. DALYs)*	*Aims of country: maintaining order in nation*	*18–23*			*0.010 2*	*0.008 05*	*0.008 05*	*−0.088 8*	*−0.025 8*
*ρ*/*β* (COVID-19)	Important for country: ideas count more than money	18–23			0.582**/0.167	0.554**/0.13	0.534**/0.093 4	0.513*/1.28*	0.499*/0.242
*β (env. DALYs)*	*Important for country: ideas count more than money*	*18–23*			*−0.063 3*	*−0.069 5*	*−0.069 5*	*−0.15*	*−0.051 4*
*ρ*/*β* (COVID-19)	Rejected neighbors: people of a different religion	18–23	−0.544*/*−*1.77	−0.679**/*−*1.8*					
*β (env. DALYs)*	*Rejected neighbors: people of a different religion*	*18–23*	*0.076*	*−0.069 8*					
*ρ*/*β* (COVID-19)	Post-materialist index (12 item)	18–23		0.59**/0.316	0.529**/0.582				
*β (env. DALYs)*	*Post-materialist index (12 item)*	*18–23*		*−0.022 7*	*−0.002 52*				

Finally, to explore the meaning of the WVS predictors identified in the steps above, I extracted those WVS predictors that were correlated both with COVID-19-related mortality and the WVS predictors above, assigned a theme to each of them, and extracted the most frequent themes (see SM1 and SM5).

As mentioned above, I sought to determine whether the WVS predictors above were specific to COVID-19-related mortality, or whether they simply predicted health outcomes in general. To do so, I repeated the steps above for a general measure of health (healthy life expectancy at age 60, sourced from the World Health Organization) as well as for a measure mortality in a domain where institutional structures are important (Disability-Adjusted-Life-Years (DALYs) lost due to environmental causes (excluding infectious, parasitic, neonatal and nutritional diseases; Prüss-Üstün et al., 2016). (DALYs indicate the number of deaths weighted by the remaining life expectancy, as well a term corresponding to the time an individual needs to live with some impairment.)

## Results

### Values Predicting COVID-19-Related Mortality

Across country sets and snapshots, the three WVS predictors most consistently associated with a *decrease* of COVID-19-related mortality were a weaker endorsement of freedom of speech as a goal for the country, a greater endorsement of maintenance of order as a goal for the country, and a greater confidence in major companies. (The WVS question about goals for the country had four response options: protecting freedom of speech, maintaining order in the nation, giving people more say, and fighting rising prices. The proportion of choosing one option is thus the proportion of *not* choosing the other options.)

Table 1 shows the WVS predictors extracted for each country set and snapshot. Adding the WVS predictors to the baseline models improved predictions for almost all snapshots and country sets, though, for some snapshots with smaller country sets (early and late snapshots for Advanced Economies; middle snapshots for High Income Economies), these improvements were not reliable in cross-validation. Depending on the country set and snapshot, the models accounted for up to 68% of the between-country variability in COVID-19-related mortality. Increased endorsement of freedom of speech or related WVS predictors (e.g., participation in demonstrations, endorsement of civil rights or unemployment benefits, and reduced endorsement of maintenance of order) were associated with increased COVID-19-related mortality across country sets; inspection of the correlation coefficients in Table 1 suggests that this variable was generally the most important predictor of COVID-19-related mortality. An apparent exception is that the importance accorded to politics was associated with *reduced* COVID-19-related mortality for Upper-Middle Income Economies. However, inspection of the pattern of intercorrelations among WVS predictors suggests that an interest in politics does not reflect an endorsement of freedom of speech. Specifically, to better understand the meaning of the WVS predictors, I extracted those WVS predictors that were correlated both with COVID-19-related mortality and the WVS predictors above.^1^ As shown in Table 2, endorsement of freedom of speech tended to be positively associated with WVS predictors reflecting gender equality, social tolerance and a less materialistic orientation. In contrast, an interest in politics tended to be *negatively* correlated with these values. The negative association between the interest in politics and COVID-19-related mortality is thus consistent with the finding that an endorsement of freedom of speech is associated with increased COVID-19-related mortality.

**Table 2.** WVS predictors that were correlated both with the selected WVS predictors from Table 1 (bold face) and COVID-19-related mortality. The correlated WVS predictors were combined across snapshots and country sets. (+) indicates a positive correlation while (−) indicates a negative correlation.

**Aims of country: protecting freedom of speech:** confidence in press (−), aims of country: giving people greater say (+), post-materialist index (12 item) (+), signed a petition (+), government (rather than people) should take responsibility (−), men make better political leaders than women (−), satisfaction with life (+), important child qualities: tolerance/respect for other people (+), important for person: thinking up new ideas and being creative (+), women have same rights as men (+), most serious problem of the world: discrimination against girls/women (+), joined unofficial strikes (+)
**Confidence in press:** important child qualities: determination and perseverance (+), aims of country: protecting freedom of speech (−), confidence in major companies (+), religious person (−), most important for country: fight against crime (−), confidence in police (+), confidence in armed forces (+), good political system: having experts make decisions (−), respect for individual human rights nowadays (+), important child qualities: tolerance/respect for other people (−), important for person: politics (+), greater respect for authority is a good thing (−), important for person: thinking up new ideas and being creative (−), important for person: always behaving properly (−), important for person: tradition (−), most serious problem of the world: people living in poverty (−), most serious problem of the world: environmental pollution (+), joined unofficial strikes (−), men make better political leaders than women (+), confidence in parliament (+), important child qualities: obedience (−), confidence in United Nations (+), justifiable: divorce (−), university is more important for boys than girls (+), important child qualities: thrift and saving money/things (+)
**Religious person:** important child qualities: determination and perseverance (−), confidence in press (−), confidence in major companies (−), most important for country: fight against crime (+), confidence in police (−), respect for individual human rights nowadays (−)
**Confidence in major companies:** aims of country: maintaining order in nation (+), most important for country: fight against crime (−), confidence in press (+), religious person (−), confidence in police (+), confidence in armed forces (+), satisfaction with life (+), respect for individual human rights nowadays (+), important for person: politics (+), greater respect for authority is a good thing (−), important for person: thinking up new ideas and being creative (−), most serious problem of the world: people living in poverty (−), joined unofficial strikes (−), confidence in parliament (+), confidence in United Nations (+), justifiable: divorce (−), important child qualities: thrift and saving money/things (+)
**Rejected neighbors:** people of a different religion: thinking about meaning and purpose of life (+)
**Signed a petition:** important child qualities: feeling of responsibility (−), satisfaction with life (−)
**Post-materialist index (12 items):** aims of country: protecting freedom of speech (+), important for country: ideas count more than money (+)
**Aims of country: maintaining order in nation:** important for country: ideas count more than money (−), important child qualities: religious faith (−), most people can be trusted (+), one of the main goals in life has been to make my parents proud (−), essential characteristics of democracy: army takes over when government is incompetent (−)
**Important for country: ideas count more than money:** aims of country: maintaining order in nation (−), post-materialist index (12 item) (+)
**Important child qualities: religious faith:** aims of country: maintaining order in nation (−), confidence in churches (+), good political system: having army rule (+), one of the main goals in life has been to make my parents proud (+)
**Important for person: politics:** important for person: thinking up new ideas and being creative (−), important for person: tradition (−), important child qualities: tolerance/respect for other people (−), important child qualities: obedience (−), interest in politics (+), aims of country: giving people greater say (−), most important for country: fight against crime (−), greater respect for authority is a good thing (−), confidence in press (+), confidence in major companies (+), confidence in United Nations (+), religious person (−), confidence in parliament (+), women have same rights as men (−), most serious problem of the world: people living in poverty (−), justifiable: divorce (−), rejected neighbors: people of a different race (+), most serious problem of the world: environmental pollution (+), good political system: having experts make decisions (−)
**Important for person: leisure time:** private (rather than government) ownership of business should be increased (+)
**Good political system: having experts make decisions:** justifiable: divorce (+), important for person: politics (−), important child qualities: obedience (+), rejected neighbors: people of a different race (−), most important for country: fight against crime (+)
**Justifiable: divorce:** important for person: politics (−), important child qualities: obedience (+), rejected neighbors: people of a different race (−), most important for country: humane society (−), most important for country: fight against crime (+), confidence in major companies (−), good political system: having experts make decisions (+), attended lawful peaceful demonstrations (+)
**Essential characteristics of democracy: people receive state aid for unemployment:** important child qualities: feeling of responsibility (+), confidence in parliament (+), aims of country: protecting freedom of speech (+), essential characteristics of democracy: civil rights protect people’s liberty against oppression (+)
**Good political system: having army rule:** important child qualities: religious faith (+), important for country: ideas count more than money (+), confidence in churches (+), essential characteristics of democracy: army takes over when government is incompetent (+)

Confidence in major companies and the press was negatively associated with COVID-19-related mortality when considering all available countries and when combining Upper-Middle and High Income Economies. As shown in Table 2, these WVS predictors were positively correlated with other WVS predictors reflecting increased trust in major institutions and reflecting reduced endorsement of freedom of speech, gender equality and social tolerance, but also of conformity.

When considering all countries and High Income Economies, WVS predictors reflecting increased religiosity also predicted increased COVID-19-related mortality. While increased religiosity was associated with reduced trust in major institutions, there were no other clear themes in the pattern of intercorrelations with other WVS predictors.

When considering Upper-Middle Income and Advanced Economies, a less materialistic orientation was also associated with increased COVID-19-related mortality. As mentioned above, the pattern for intercorrelations among WVS predictors shows that a less materialistic orientation is associated with an increased endorsement of freedom of speech.

For Upper-Middle Economies, endorsement of technocratic governments was associated with increased COVID-19-related mortality. As shown in Table 2, this WVS predictor correlated with values reflecting increased social tolerance, but also a reduced endorsement of authority.

To explore the meaning of these WVS predictors, I extracted those WVS predictors that were correlated both with COVID-19-related mortality and the WVS predictors above, assigned a theme to each of them, and extracted the most frequent themes (see SM1 and SM5). The most frequent theme associated with reduced COVID-19-related mortality was confidence in major institutions. The most frequent themes associated with increased COVID-19-related mortality were WVS predictors reflecting social tolerance, gender equality, democratic or political participation, a more post-materialistic or hedonic orientation, religiosity, but also an increased focus on crime and greater acceptance of authority and conformity.

**Figure 1.**
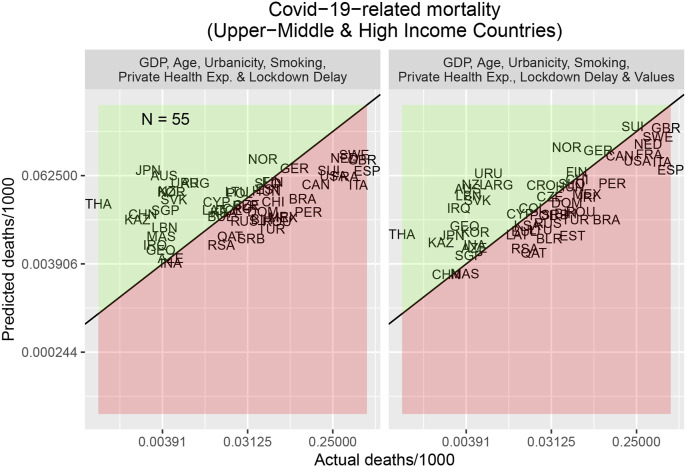
Predicted and actual COVID-19-related mortality for Upper-Middle and High Income Economies for a snapshot 70 days after the first confirmed COVID-19-related death in each country. Country acronyms reflect IOC codes. Green and red areas signal smaller or greater than expected mortalities, respectively. (Left) Results for the baseline model including only covariates (see main text). (Right) Results for the full model including the WVS predictors (see Table 1 for the predictors).

As an illustration, Figure 1 shows predicted and actual COVID-19-related mortalities for Upper-Middle and High Income Economies 70 days after the first COVID-19-related death in each country. (Similar figures for the other country sets are given in SM2.) Countries like Canada and Switzerland have higher COVID-19-related mortalities than expected from the covariates above, but are predicted to have higher mortalities when including their values. Conversely, countries like Japan and South Korea have lower COVID-19-related mortalities than expected from the aforementioned covariates, but are predicted to have lower mortalities when including their values.

### Values Predicting Other Health Outcomes

I next asked if these WVS predictors were specific to COVID-19-related mortality, or whether they predicted health in general. I thus sought to predict a general measure of health (healthy life expectance at age 60; HALE-60) based on WVS predictors. However, the predictors of COVID-19-related mortality did not improve prediction errors for HALE-60 compared to the baseline model (except when all countries are considered). Further, for most country sets (except for Advanced Economies), I could not even identify WVS predictors of HALE-60 using the criteria above.

Socio-cultural values thus do not seem to predict health in general. A possible explanation of this finding is that different socio-cultural values might affect different health outcomes in different ways, and might even have opposite effects. To test this possibility, I sought to predict mortality in a domain where institutional structures are important in addition to individual behavior: Disability-Adjusted-Life-Years (DALYs) lost due to environmental causes (excluding infectious, parasitic, neonatal and nutritional diseases; Prüss-Üstün et al., 2016). As shown in Table 1 (gray rows), WVS predictors of COVID-19-related mortality did improve predictions of environmental DALYs numerically or significantly for many snapshots and country sets. Critically, however, the predictor most consistently associated with both types of mortality—endorsement of freedom of speech—had opposite signs: Greater endorsement for freedom of speech was associated with increased COVID-19-related mortality, but decreased environmental mortality.

To confirm this impression, I extracted WVS predictors of environmental mortality for the country sets used 70 days after the first COVID-19-related death in each country. Even though environmental DALYs encompass a fairly diverse set of conditions, the models accounted for up to 90% of across-country variability in environmental mortality and were reliable in cross-validation for all country sets (see Table 3). While the specific predictors differed across country sets, reduced environmental mortality was associated with an endorsement of social tolerance and gender equality and a less materialistic orientation and thus with values that predict *increased* COVID-19-related mortality. This was confirmed by considering the themes of the WVS predictors that were correlated with both the extracted WVS predictors and environmental-related mortality. The most frequent themes associated with a reduction in environmental mortality were a more post-materialistic or hedonic orientation, endorsement of democratic or political participation, gender equality and social tolerance, a greater feeling of self-direction and greater life satisfaction, and thus, values that are associated with *increased* COVID-19-related mortality, though the environmental-related WVS predictors did not reliably improve predictions of COVID-19-related mortality. Be this at this may, these results suggest that the WVS predictors associated with COVID-19-related mortality are relatively specific to that health outcome.

**Table 3.** WVS predictors of environmental-related DALYs and results from a linear model predicting environmental DALYs (middle) and COVID-19-related mortality (right). See Table 1 for definitions. The models below did not use the lockdown delay as a covariate. See Table S3 for similar analyses in which government effectiveness and the rule of law are included as covariates.

	Correlation			Environmental model				COVID-19 model			
Predictor	*N*	*ρ*	*p*	*β*	*SE*	*t*	*p*	*β*	*SE*	*t*	*p*
**Predictors for environmental-related DALYs (all countries, *N* = 79,**${\bar{R}}^{2}$**= 0.711)**											
Important child qualities: hard work	82	0.475	0.000	0.088	0.031	2.819	0.006	−0.289	0.213	−1.354	0.180
Rejected neighbors: people with aids	79	0.388	0.000	0.054	0.036	1.510	0.136	−0.166	0.244	−0.680	0.499
Rejected neighbors: drug addicts	79	0.385	0.000	0.024	0.032	0.741	0.461	0.107	0.220	0.489	0.627
***% reduction prediction error* versus *baseline***				14.65*** (full model), 8.79*** (cross validation)				*1.92 (full model)*			
**Predictors for environmental-related DALYs (upper-middle and high income economies, *N* = 44,**${\bar{R}}^{2}$**= 0.766)**											
Rejected neighbors: drug addicts	56	0.474	0.000	0.047	0.043	1.110	0.274	0.128	0.340	0.377	0.709
Science and technology are making lives healthier easier and more comfortable	45	0.454	0.002	0.089	0.051	1.740	0.091	0.018	0.409	0.045	0.964
Men make better political leaders than women	57	0.446	0.001	0.124	0.070	1.766	0.086	−0.554	0.561	−0.987	0.330
***% reduction prediction error* versus *baseline***				22.09*** (full model), 14.57*** (cross validation)				*1.68 (full model)*			
**Predictors for environmental-related DALYs (upper-middle income economies, *N* = 18,**${\bar{R}}^{2}$**= 0.852)**											
Important child qualities: hard work	24	0.653	0.001	0.089	0.104	0.855	0.415	1.919	0.888	2.160	0.059
Most serious problem of the world: inadequate education	21	−0.624	0.002	−0.146	0.063	−2.301	0.047	0.643	0.542	1.186	0.266
Participated in demonstration for environment in past 2 years	18	−0.614	0.007	−0.089	0.074	−1.210	0.257	0.730	0.633	1.154	0.278
***% reduction prediction error* versus *baseline***				48.32** (full model), 14.6*** (cross validation)				*21.62 (full model)*			
**Predictors for environmental-related DALYs (high income economies, *N* = 31,**${\bar{R}}^{2}$**= 0.904)**											
Proud of nationality	32	−0.537	0.002	−0.118	0.034	−3.447	0.002	−0.478	0.423	−1.129	0.271
One of the main goals in life has been to make my parents proud	31	−0.528	0.002	−0.098	0.040	−2.448	0.023	0.941	0.492	1.914	0.069
Important child qualities: unselfishness	32	−0.526	0.002	−0.071	0.025	−2.803	0.011	−0.170	0.313	−0.543	0.593
***% reduction prediction error* versus *baseline***				43.54*** (full model), 34.02*** (cross validation)				*9.7 (full model)*			
**Predictors for environmental-related DALYs (advanced economies, *N* = 22,**${\bar{R}}^{2}$**= 0.893)**											
Proud of nationality	23	−0.666	0.001	−0.094	0.037	−2.533	0.026	−0.356	0.566	−0.629	0.541
Self-positioning on political right	22	0.661	0.001	0.059	0.024	2.522	0.027	−0.272	0.360	−0.754	0.465
Important child qualities: unselfishness	23	−0.651	0.001	−0.046	0.028	−1.652	0.125	0.323	0.431	0.750	0.468
***% reduction prediction error* versus *baseline***				50.86*** (full model), 31.14*** (cross validation)				*7.36 (full model)*			

## Discussion

The current results suggest that COVID-19-related mortality is increased in societies that value democratic or political participation, social tolerance, gender equality, and have a more post-materialistic or hedonic orientation. In contrast, societies that place more confidence in major institutions have reduced COVID-19-related mortalities. These risk factors were reliable despite controlling for health-related covariates that likely reflect the socio-cultural values of a society (e.g., the proportion of-out-of-pocket health expenditure and the delay of the first government COVID-19-related containment measure), though not controlling for these covariates leads to very similar results; including government effectiveness and the rule of law as covariates yielded similar results as well. Critically, while it is still possible that mortality might be confounded with differences in public healthcare systems, such differences likely reflect the values in a society, given that, at least historically, public health spending depended on government ideology (Herwartz & Theilen, 2013; Potrafke, 2010).

While previous investigations have linked COVID-19-related outcomes to established constructs such as political affiliation, social capital or cultural tightness (e.g., Gelfand et al., 2021; Grossman et al., 2020; Hsiehchen et al., 2020; Makridis & Wu, 2021; Min, 2020, but see Romano et al., 2021), the specificity of these associations is unclear. The current results show that these risk factors are relatively specific to COVID-19-related mortality, as they do not predict health in general. The current results also show why these risk factors do not predict general health outcomes, as they are associated with *decreased* mortality due to other health outcomes such as environmental mortality. Presumably, values such as an endorsement of freedom of speech and a less materialistic orientation are conducive to investigating and addressing environmental issues, even though they are risk factors for COVID-19-related mortality. However, the specific causal pathways still need to be elucidated (see below). Be that as it might, the current results suggest that it might be feasible to use different sets of societal values as relatively specific risk factors for specific health outcomes.

### How Do Specific Values Map Onto Cultural Dimensions?

The existence of societal values predicting mortality raises the question of how these values relate to cultural dimensions proposed in earlier work (e.g., Hofstede, 1980; Inglehart, 1997; Inglehart & Welzel, 2005; Schwartz, 2004). As mentioned above, due to the relatively sparse dataset, I could not use dimensionality reduction techniques (e.g., principal component analysis) without sacrificing coverage either in the number of countries or the number of predictors. However, inspection of Table 2 suggests that in Inglehart’s (1997) and Inglehart and Welzel’s (2005) classification (that is also based on the WVS), survival-related (rather than self-expression) values have a protective effect against COVID-19-related mortality. While the values related to political participation are not included in their analyses, Kaasa’s (2021) alignment of the WVS values with those developed by Hofstede (1980) and Schwartz (2004) suggests that values related to political participation (i.e., power closeness and egalitarianism) map onto both secular-rational authority (rather than traditional) values and, to some extent, survival-related (rather than self-expression) values. These results thus confirm in a more data-driven analysis that COVID-19-related mortality is reduced in societies with more survival-related values and traditional authorities. This also confirms earlier results suggesting that “tighter” cultures with more stringent social norms have reduced COVID-19 mortality (Gelfand et al., 2021); as societies that have historically faced more environmental or human-made threats tend to have stronger social norms and lower tolerance of non-conforming behavior (Gelfand et al., 2011; Harrington & Gelfand, 2014; see also Inglehart, 1997 for a related point), historic cultural adaptation to biological threats might also be effective for novel threats.

However, it is unclear to what extent the confidence in major institution maps on any of these dimensions; in fact, inspection of the pattern of correlations Table 2 suggests that confidence in institutions does not map onto any of these dimensions. It is thus possible that confidence in institutions is a predictor of COVID-19-related mortality that is relatively independent of the other factors, though the extent of its independence needs to be established in more targeted studies.

In contrast, comparison of Table 4 with Inglehart (1997, p. 82) and Inglehart and Welzel (2005, p. 49) suggests that survival-related values as well as traditional forms of authority are associated with *increased* environmental-related mortality. To the extent that such cultural values reflect historic environmental stress (Gelfand et al., 2011; Harrington & Gelfand, 2014), such historic adaptations might be maladaptive for contemporary threats.

**Table 4.** WVS predictors that were correlated both with the selected WVS predictors from Table 3 (bold face) and environmental-related mortality. The correlated WVS predictors were combined across snapshots and country sets. (+) indicates a positive correlation while (−) indicates a negative correlation.

**Proud of nationality:** confidence in press (−), feeling of happiness (+), feeling of freedom of choice and control over life (+), important child qualities: unselfishness (+), important for person: friends (+), most people can be trusted (−), rejected neighbors: drug addicts (−), satisfaction with life (+), important for person: adventure/taking risks (+)
**Important child qualities:** hard work: membership of sport/recreation (−), aims of country: economic growth (+), aims of country: protecting freedom of speech (−), government (rather than people) should take responsibility (+), I see myself as an autonomous individual (−), importance of democracy (−), important child qualities: imagination (−), important child qualities: unselfishness (−), important for person: work (−), men make better political leaders than women (+), most important for country: humane society (−), most serious problem of the world: inadequate education (−), rejected neighbors: drug addicts (+), rejected neighbors: heavy drinkers (+), rejected neighbors: people with aids (+), participated in demonstration for environment in past 2 years (−), post-materialist index (12 item) (−)
**Important child qualities: unselfishness:** feeling of happiness (+), most people can be trusted (−), justifiable: accepting a bribe (−), proud of nationality (+), self-positioning on political right (−), confidence in press (−)
**Men make better political leaders than women:** feeling of happiness (−), important child qualities: hard work (+), important for person: leisure time (−), important for person: work (−), aims of country: maintaining order in nation (+), aims of country: protecting freedom of speech (−), increased emphasis on technology is a good thing (+), justifiable: homosexuality (−), rejected neighbors: drug addicts (+), being a housewife just as fulfilling (+), membership of religious organization (−), membership of sport/recreation (−), good political system: having a strong leader (+), important for person: having good time (−), the world is better off because of science and technology (+), most serious problem of the world: inadequate education (−), science and technology are making lives healthier easier and more comfortable (+), joined unofficial strikes (−)
**Most serious problem of the world:** inadequate education: important child qualities: hard work (−), government (rather than people) should take responsibility (−), trust people you meet for the first time (−), social position of people in their 40s (−)
**Rejected neighbors:** drug addicts: membership of religious organization (−), aims of country: protecting freedom of speech (−), being a housewife just as fulfilling (+), feeling of happiness (−), important child qualities: hard work (+), important for person: work (−), justifiable: homosexuality (−), men make better political leaders than women (+), most serious problem of the world: inadequate education (−), rejected neighbors: people with aids (+), signed a petition (−), good political system: democracy (−), good political system: having a strong leader (+), post-materialist index (12 item) (−), important for person: having good time (−), science and technology are making lives healthier easier and more comfortable (+), the world is better off because of science and technology (+)
**Rejected neighbors:** people with aids: important child qualities: hard work (+), important child qualities: imagination (−), men make better political leaders than women (+), aims of country: protecting freedom of speech (−), post-materialist index (12 item) (−), rejected neighbors: drug addicts (+), good political system: having a strong leader (+), good political system: democracy (−), membership of sport/recreation (−), signed a petition (−)
**One of the main goals in life has been to make my parents proud:** less importance placed on work is a good thing (+), self-positioning on political right (−)
**Participated in demonstration for environment in past 2 years:** important child qualities: hard work (−), trust people you meet for the first time (−), social position of people in their 40s (−)
**Science and technology are making lives healthier easier and more comfortable: important child qualities:** hard work (+), men make better political leaders than women (+), aims of country: economic growth (+), aims of country: protecting freedom of speech (−), justifiable: homosexuality (−), rejected neighbors: drug addicts (+), membership of religious organization (−), membership of sport/recreation (−), important for person: having good time (−), important for person: adventure/taking risks (−), essential characteristics of democracy: civil rights protect people’s liberty against oppression (+), the world is better off because of science and technology (+), joined unofficial strikes (−)
**Self-positioning on political right:** important child qualities: unselfishness (−), most people can be trusted (+), confidence in press (+)

### Potential Causal Pathways

Societal values might affect COVID-19-related mortality in at least four potentially interacting ways, depending on whether they affect health outcomes via behavioral patterns in the general population, via behavioral patterns and beliefs by the individuals carrying out government action, or via institutional structures.

First, societal values might promote behaviors in the general population that affect COVID-19-related outcomes. There is some support for this possibility. For example, political attitudes predict compliance with COVID-19 containment measures (Grossman et al., 2020; Hsiehchen et al., 2020); more generally, societies with more survival-related values, traditional authorities and tighter cultural norms might be more likely to adhere to public health guidance. Likewise, it is at least plausible that individuals with greater trust in institutions will also be more likely to trust (and thus comply with) public health guidance.

Second, governments are run by humans, who are affected by the values of the society they are embedded in. As a result, societal values might lead the individuals embodying governments to decisions that affect COVID-19-related outcomes. There is some support for this possibility as well. For example, governments in societies with stronger democratic norms were slower to enact stringent containment measures (Sebhatu et al., 2020), which likely leads to increased mortality.

Third, and relatedly, *perceptions* of societal values might guide the actions and decisions of the individuals embodying governments. For example, they might take those containment measures they deem acceptable for their society, that are considered likely to be followed or that promise electoral success (where applicable), which is also consistent with the finding that governments in societies with stronger democratic norms were slower to enact stringent containment measures (Sebhatu et al., 2020).

Fourth, societal values presumably shape the institutional structures in a society, which might affect health outcomes. There is some support for this possibility as well. For example, while the analyses presented here control for the need for private health expenditures, public health spending depends on historic government ideology (Herwartz & Theilen, 2013; Potrafke, 2010), and greater healthcare capacity reduces COVID-19-related mortality (Khan et al., 2020, but see Acharya et al., 2021).

Importantly, while these causal pathways might reinforce each other (e.g., when government decisions reinforce population-level behavioral biases), they might also be independent of one another. For example, collective behavior might be more responsive to government measures than anticipated by the individuals embodying the government, and collective behavior might thus be affected by *perceptions* of societal values. However, given that the values of governments (presumably) reflect the values of their society at least to some extent, these causal pathways are difficult to disentangle in the absence of targeted intervention studies. It is thus an important question for future research to find out which societal values create collective behavioral biases that affect health, under which conditions societal values encourage or impede effective health interventions, and to what extent government responses to disease are independent of (the perception of) societal values.

These causal pathways also explain why socio-cultural values have different effects on different health outcomes. For example, compliant individual behavior might help reducing the spread of COVID-19, but might make it harder to confront environmental pollution, especially when committed by powerful entities. Likewise, trust in institutions might encourage effective health-related behaviors; however, it might also make it easier for public institutions not to confront environmental pollution and also to commit it in the first place. The same societal values might thus have different effects on different health outcomes, suggesting that such values might be relatively specific risk factors for health outcomes.

### Methodological Implications

The finding that the same socio-cultural values can have different effects on different health outcomes has both methodological and practical implications. Methodologically, comparing the effects of a set of predictors on different health outcomes serves as an additional safeguard for the validity of the predictors. This is important because it is difficult to find all potentially relevant confounding variables. If the predictors have different effects for different outcome variables, one can be more confident that they are specifically associated with the outcome variables, rather than being associated with some more general variable such as general health.

Practically, the current results highlight that interventions can have unintended consequences. For example, to the extent that the associations reported here reflect causal links, an intervention increasing support for political participation might be beneficial in terms of environmental-related mortality, but might have detrimental consequences for mortality due to infectious disease.

### Limitations

There are several caveats regarding the reliability of the data and their interpretation. First, mortality data relies on the capability and willingness to accurately report the data, which depends on societal values. However, as the pattern of associations was relatively consistent across country sets and snapshots, and generally also in cross-validation, the current results are unlikely to rely exclusively on misreporting by some countries.

Second, there is geographical variation that the models do not control for and that might affect susceptibility to infectious disease. For example, some countries are islands, and might plausibly be better able to isolate themselves. However, islands also tend to be smaller, more open to external trade, and more dependent on tourism (e.g., Armstrong et al., 1998; Armstrong & Read, 2020). This might make infectious diseases more likely to reach an island and subsequently spread. As a result, it is not clear which correlates of insularity would be the most relevant risk factors for infectious disease, and whether insularity affects COVID-19-related mortality. For example, two island countries such as the United Kingdom and New Zealand differ in their COVID-19-related mortality by a factor of more than 200.

Relatedly, while I controlled for the proportion of the population in urban areas, countries differ in their typical city layouts and generational co-residence patterns, which might affect mortality due to infectious disease. However, the effects of co-residence patterns on COVID-19-related mortality are controversial (e.g., Arpino et al., 2020; Dowd et al., 2020; Esteve et al., 2020). Further, including such factors as covariates in the current value-based predictions would require sub-national data about values that are not available.

Third, there is substantial within-country heterogeneity in cultural norms. For example, even in a single country, political attitudes predict compliance with COVID-19 containment measures (Grossman et al., 2020; Hsiehchen et al., 2020); given that conservative political leanings are associated with cultural tightness (Harrington & Gelfand, 2014), these results seem inconsistent with the cross-national result that cultural tightness is associated with reduced COVID-19-related mortality (Gelfand et al., 2021). Be that as it may, given that the association between values and mortality was found despite this heterogeneity, value-based predictions might improve for smaller demographic units.

Fourth, it is unclear how the WVS predictors should be interpreted and whether respondents in all countries understand values in the same way. For example, Algeria and Estonia are among the countries placing the highest and lowest importance on freedom of speech (*Z* = 0.893 and −1.262, respectively)—even though they are ranked 146th and 14th (out of 180) for press freedom (Word Press Freedom Index 2020). As my models did not include interaction terms, the role or meaning of WVS predictors might be different in different countries. Relatedly, most analyses focused on countries that had reached at least the Upper-Middle Income stage, and the predictors for poorer countries might well be different from those in their wealthier counterparts. However, given that the results for the *all countries* country set (comprising an additional 20 countries) yielded rather similar results to those for the wealthier countries, it seems likely that the values predicting mortality are not substantially different in poorer countries.

Fifth, COVID-19-related mortality likely depends on government responses, but the government responses likely depend on societal values in turn. In fact, endorsement of freedom of speech as a goal for the country was correlated with the (pre-COVID-19) 2019 Economist Democracy Index (The Economist Intelligence Unit, 2020).^2^ Democratic political systems might thus thrive because individuals support freedom of speech, or individuals in democratic societies might support freedom of speech because they live in democratic countries allowing its expression to begin with. This makes it difficult to describe specific causal pathways by which societal values might affect COVID-19-related outcomes. In fact, as mentioned above, the present results suggest several possible causal pathways that can only be disentangled through targeted studies.

## Conclusions

Taken together, the current results demonstrate that a limited set of socio-cultural values substantially improves prediction accuracy for different health outcomes. This association raises the question of causality. Some values reflect historical environmental conditions that, in some cases, even predate modern humans (Hauser, 2006). For example, and as mentioned above, societies that have historically faced more environmental or human-made threats tend to have stronger social norms and lower tolerance of non-conforming behavior (Gelfand et al., 2011; Harrington & Gelfand, 2014). Such values might *create* new health risks in contemporary environments, and behavioral interventions targeting such values might improve health outcomes. Other values might reflect current environments. For example, societies with trustworthy institutions might have confidence in these institutions. Such values might be real-time indicators of health risks. For example, the loss of confidence in institutions might signal or cause increased mortality, similarly to how (online) behavior has been used to track the prevalence of other infectious diseases (Ginsberg et al., 2009; Brooks et al., 2015). It is thus important to address the cause–effect relationship between values and health outcomes and to ask whether values help predicting other health outcomes.

## Notes

1. An alternative, and mathematically equivalent, interpretation of this approach is to first identify WVS items that are correlated with the selected WVS predictors, and then to test whether these items are correlated with COVID-19 mortality as well.

2. Full democracies: *ρ* = 0.442, *p* = 0.116, ${\bar{R}}^{2}$ = 0.104 (*N* = 14); full and flawed democracies: *ρ* = 0.397, *p* = 0.004, ${\bar{R}}^{2}$ = 0.278 (*N* = 51); full and flawed democracies, hybrid regimes: *ρ* = 0.389, *p* = 0.001, ${\bar{R}}^{2}$ = 0.231 (*N* = 72); all countries: *ρ* = 0.429, *p* = < .001, ${\bar{R}}^{2}$ = 0.239 (*N* = 91).
